# Association of breast cancer with MRI background parenchymal enhancement: the IMAGINE case-control study

**DOI:** 10.1186/s13058-020-01375-7

**Published:** 2020-12-07

**Authors:** Gordon P. Watt, Janice Sung, Elizabeth A. Morris, Saundra S. Buys, Angela R. Bradbury, Jennifer D. Brooks, Emily F. Conant, Susan P. Weinstein, Despina Kontos, Meghan Woods, Sarah V. Colonna, Xiaolin Liang, Matthew A. Stein, Malcolm C. Pike, Jonine L. Bernstein

**Affiliations:** 1 Department of Epidemiology and Biostatistics, Memorial Sloan Kettering Cancer Center, 485 Lexington Ave., Second Floor, New York, NY 10017 USA; 2 Department of Radiology, Memorial Sloan Kettering Cancer Center, New York, USA; 3 Huntsman Cancer Institute, University of Utah, Salt Lake City, USA; 4 Department of Medicine, Perelman School of Medicine at the University of Pennsylvania, Philadelphia, USA; 5 Dalla Lana School of Public Health, University of Toronto, Toronto, Canada; 6 Department of Radiology, Perelman School of Medicine at the University of Pennsylvania, Philadelphia, USA

**Keywords:** Breast cancer, Background parenchymal enhancement, Magnetic resonance imaging, Risk factors, Case-control study

## Abstract

**Background:**

Background parenchymal enhancement (BPE) on breast magnetic resonance imaging (MRI) may be associated with breast cancer risk, but previous studies of the association are equivocal and limited by incomplete blinding of BPE assessment. In this study, we evaluated the association between BPE and breast cancer based on fully blinded assessments of BPE in the unaffected breast.

**Methods:**

The Imaging and Epidemiology (IMAGINE) study is a multicenter breast cancer case-control study of women receiving diagnostic, screening, or follow-up breast MRI, recruited from three comprehensive cancer centers in the USA. Cases had a first diagnosis of unilateral breast cancer and controls had no history of or current breast cancer. A single board-certified breast radiologist with 12 years’ experience, blinded to case-control status and clinical information, assessed the unaffected breast for BPE without view of the affected breast of cases (or the corresponding breast laterality of controls). The association between BPE and breast cancer was estimated by multivariable logistic regression separately for premenopausal and postmenopausal women.

**Results:**

The analytic dataset included 835 cases and 963 controls. Adjusting for fibroglandular tissue (breast density), age, race/ethnicity, BMI, parity, family history of breast cancer, *BRCA1*/*BRCA2* mutations, and other confounders, moderate/marked BPE (vs minimal/mild BPE) was associated with breast cancer among premenopausal women [odds ratio (OR) 1.49, 95% CI 1.05–2.11; *p* = 0.02]. Among postmenopausal women, mild/moderate/marked vs minimal BPE had a similar, but statistically non-significant, association with breast cancer (OR 1.45, 95% CI 0.92–2.27; *p* = 0.1).

**Conclusions:**

BPE is associated with breast cancer in premenopausal women, and possibly postmenopausal women, after adjustment for breast density and confounders. Our results suggest that BPE should be evaluated alongside breast density for inclusion in models predicting breast cancer risk.

## Background

One of the strongest known risk factors of breast cancer, breast density [i.e., the “amount” of fibroglandular tissue (FGT)], is assessed using mammography and has been successfully incorporated into models of breast cancer risk [1, 2]. Breast density can also be estimated by assessing FGT on breast magnetic resonance imaging (MRI) [3]. After administration of the contrast agent during a breast MRI examination, FGT enhances to varying degrees, a phenomenon known as background parenchymal enhancement (BPE). In clinical practice, radiologists qualitatively categorize FGT as almost entirely fat, scattered, heterogeneous, or extreme, and BPE as minimal, mild, moderate, or marked, according to the Breast Imaging Reporting and Data System (BI-RADS) [4]. BPE differs widely between women and is sensitive to endogenous hormonal changes, particularly menopause [5–10], as well as exogenous factors, including menopausal hormone therapy, tamoxifen, aromatase inhibitors, radiation therapy, and chemotherapy [7, 11–13].

Previous studies have reported associations between BPE and breast cancer, with varying conclusions [14–21]. Interpretation of these studies has been limited by (A) lack of blinding to the MRI of the affected breast of women with breast cancer; (B) lack of blinding to patient clinical characteristics and history; (C) inter-reader variability in BPE assessment; and/or (D) no accounting for menopausal status, which strongly affects BPE. In contrast to these previous studies, the Imaging and Epidemiology (IMAGINE) study (*N* = 1798) employed a fully blinded and centralized approach to measure BPE—which reduced bias, eliminated inter-observer variability, and improved internal validity—in order to accurately assess the magnitude of association between BPE and breast cancer. Most importantly, the study radiologist was blinded to all clinical characteristics and case-control status and was able to visualize only one breast of each MRI series: the unaffected breast for cases and a corresponding breast for controls.

If BPE is confirmed as a reproducible marker of breast cancer risk, it will improve risk prediction and permit further personalization of breast cancer screening.

## Methods

The IMAGINE study is a multicenter, hospital-based, case-control study that recruited participants between November 2014 and September 2017. The source population included women aged ≥ 21 to < 70 years with a bilateral breast MRI between 2010 and 2017 available at breast MRI clinics at three National Cancer Institute-designated comprehensive cancer centers: Memorial Sloan Kettering Cancer Center in New York, New York (MSK); Perelman Center for Advanced Medicine at the University of Pennsylvania in Philadelphia, Pennsylvania; and Huntsman Cancer Institute at the University of Utah in Salt Lake City, Utah. Indication for MRI was not captured and included women undergoing follow-up for a suspicious lesion, referrals for treatment or second opinions at one of the IMAGINE recruitment sites, diagnostic workup, or annual screening MRI for high-risk women. Potentially eligible participants were identified both prospectively (approached at the time of their breast MRI) and retrospectively (contacted after their breast MRI). Potential breast cancer cases received a diagnosis on or after January 1, 2010, of a unilateral invasive breast cancer and/or a unilateral ductal carcinoma in situ (DCIS) and received a screening or diagnostic bilateral breast MRI prior to radiation therapy or any systemic therapy. Potential controls received a screening or follow-up bilateral breast MRI and had no history of invasive cancer or DCIS at the time of or within 6 months after the MRI. Cases and controls were ineligible if, at the time of MRI, they (a) had a previous diagnosis of any invasive cancer; (b) had a history of prophylactic mastectomy; (c) had a history of pre-pectoral breast implants; (d) had a history of breast reduction surgery; (e) had taken tamoxifen, aromatase inhibitors, or raloxifene in the preceding three months; (f) were pregnant or breast feeding in the preceding six months; or (g) were unable to speak and read English. Women were not excluded due to history of breast biopsy or fine needle aspiration. Timing of menstrual cycle at MRI date is not associated with BPE [22, 23] and was not recorded in this study. At all sites, the same multimodality recruitment approaches (in-person at breast MRI clinic, via mail, and via email) were used to contact potentially eligible women. The study was approved by the institutional review boards at each recruitment site.

After contacting potentially eligible women, participants provided informed consent and completed an epidemiological questionnaire to capture detailed family history of breast cancer, *BRCA1* and *BRCA2* testing history and mutation status, reproductive history, medical history, and demographic data. Study questionnaire data were managed using REDCap electronic data capture tools hosted at MSK [24, 25]. Study staff accessed participant medical records to confirm eligibility, abstract tumor characteristics of cases, and obtain MRI series. Final case-control status was determined 6 months after enrollment in the study to verify that women identified as controls did not develop an invasive cancer in the intervening period. Clinical MRIs were acquired using standard protocols for each institution, which included both axial and sagittal images using either 1.5- or 3-Tesla coils.

Prior to MRI characterization, controls were individually matched 1:1 to cases by race/ethnicity, recruitment site, age at MRI (within 5 years), and menopausal status at MRI. However, in order to improve the efficiency of study recruitment, cases and controls were enrolled in the IMAGINE study prior to confirming a suitable match, resulting in a number of unmatched cases and controls. Each matched case-control MRI pair was included together in a batch of 20 MRIs to be read on the same day by a single board-certified breast radiologist with 12 years’ experience (JS). For matched cases, study staff selected the unaffected breast for assessment of FGT and BPE. For matched controls, the laterality corresponding to that of the matched case was selected for assessment. For unmatched cases, the unaffected breast was assessed as usual and, for unmatched controls, the breast to be assessed was selected at random by study staff. The MRI series of the selected breast was provided to the study radiologist with the unselected breast obscured from view. The study radiologist was also blinded to case-control status and all clinical and demographic information. Following BI-RADS reporting guidelines, FGT was characterized as almost entirely fat, scattered, heterogeneous, or extreme using the T1-weighted non-fat-saturated series and BPE was estimated as minimal, mild, moderate, or marked using the T1-weighted fat-saturated sequence from the pre-contrast and the first post-contrast series along with the subtraction image [4]. Breast size was not estimated. The time from pre-contrast to the first post-contrast image may have varied slightly between institutions over the seven-year recruitment period, but the exact timing was not available. The study team also included repeat images for 130 women in selected batches of MRIs in order to assess the reproducibility of FGT and BPE by the study radiologist. The radiologist was aware of the study design but did not know which batches contained repeat images or how many repeats were included. We calculated Cohen’s weighted kappa statistics and 95% CIs for each measure [26].

All analyses were stratified by menopausal status, as BPE is known to decline sharply after menopause [8], with most postmenopausal women having minimal BPE [5]. Postmenopausal women included those whose menstrual cycles had stopped naturally at least 12 months prior to the MRI, those with a history of bilateral oophorectomy, and those who reported being postmenopausal without further details. In addition, we categorized women with a history of simple hysterectomy who were 50 years or older at time of the MRI as postmenopausal. The remaining women were considered premenopausal.

Multivariable conditional logistic regression models were used to estimate the association between BPE and breast cancer, separately for premenopausal and postmenopausal women. For premenopausal women, we adjusted for FGT (heterogeneous/extreme vs fatty/scattered), history of simple hysterectomy (yes vs no), BMI (< 25, ≥ 25 and < 30, ≥ 30 kg/m^2^), parity (nulliparous vs 1, 2, or 3 or more live births), as well as high-risk indications for breast cancer: 1st-degree female family history or 1st- or 2nd-degree male family history of breast cancer (yes vs no), *BRCA1* and *BRCA2* testing history and presence of *BRCA1* or *BRCA2* mutations (not tested vs positive vs negative), history of lobular carcinoma in situ (LCIS; yes vs no), and history of benign breast disease. The adjustment for high-risk indications accounts for the differing distribution of risk factors between controls, who were primarily high-risk women undergoing screening MRI, and cases, who represented a more average breast cancer risk population. We further accounted for the matched study design by conditioning the multivariable models on matching criteria: race/ethnicity (non-Hispanic White vs other), recruitment site, and age at MRI (5-year categories). The adjustment for matching variables controls for selection bias inherent in matched case-control studies [27]. Models of postmenopausal women were further adjusted for history of bilateral oophorectomy (yes vs no). In an exploratory analysis, based on a previous study reporting an association between reductions in adipose tissue and BPE [28], BMI-stratified models (BMI < 25 vs BMI ≥ 25) were estimated to evaluate effect modification by BMI.

Among cases, we evaluated the association of BPE (in the unaffected breast) with the patient’s tumor characteristics: hormone (estrogen and progesterone) receptors, human epidermal growth factor 2 (HER2) expression, histological subtype (ductal, lobular, mixed, or other), and stage. In multivariable logistic regression models separately for premenopausal and postmenopausal women, we regressed BPE on these factors with additional adjustment for FGT, BMI, age, recruitment site, and race/ethnicity. All analysis was conducted in R version 3.5.1 [28]. Two-sided statistical significance was set at 5%.

## Results

**Fig. 1:**
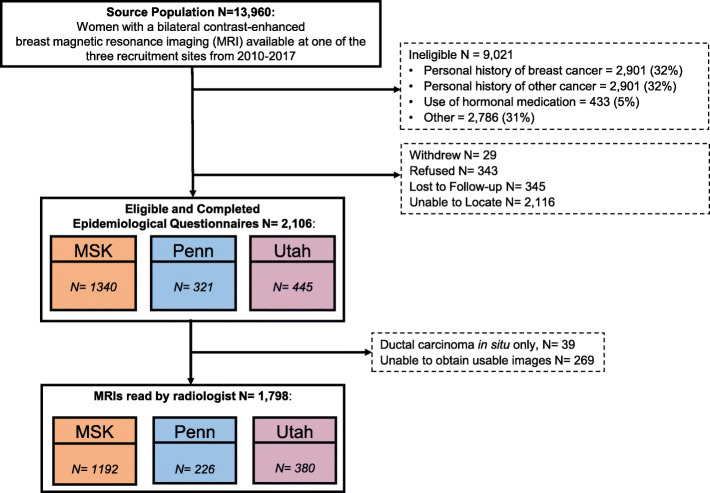
Participants in the IMAGINE study were recruited from women undergoing breast MRI from three National Cancer Institute-designated Comprehensive Cancer Centers between 2010 and 2017. The final analytic sample included 1798 women with a valid assessment by a single board-certified radiologist

Among 13,960 women with bilateral contrast-enhanced breast MRI at one of the recruitment sites between 2010 and 2017, 9021 were ineligible (64% were ineligible due to previous cancer diagnosis) and 343 refused (102 confirmed cases and 64 confirmed controls, others not assigned). There were 2106 women who consented, completed the epidemiological questionnaire, and provided access to medical records and MRI series. Twenty-nine women (4 confirmed cases and 10 confirmed controls, others not assigned) ultimately withdrew from the study. We excluded 39 cases who had DCIS without invasive cancer and 269 women with MRI series that were either corrupted or deemed inadequate for accurate assessment of FGT and BPE by the study radiologist, leaving a total of 1798 women (835 cases 963 controls) in the analytic dataset with complete MRI assessments for analysis. In Fig. 1, we provide additional details of study recruitment. The median time between MRI and questionnaire was 10 days [interquartile range (IQR) 0–240] for controls and 233 days (IQR 41–979) for cases.

Table 1 displays the characteristics of the study population. Control women were more likely than cases to have a family history of breast cancer, *BRCA1* and *BRCA2* mutations, a history of LCIS and a history of benign breast disease. Therefore, in the multivariable models, we adjusted for these differences between cases and controls. Among cases, tumors were primarily ductal and confined to the breast. There were 168 participants (20 cases and 148 controls) without a match for MRI assessment. Women without a match were more likely to be recruited from MSK and were younger at the time of their MRI but otherwise did not differ notably from the matched women (Supplementary Table 1).

**Table 1:** Characteristics of the IMAGINE study participants

	Premenopausal women		Postmenopausal women	
	Cases	Controls	Cases	Controls
	***N*** = 553	***N*** = 623	***N*** = 282	***N*** = 340
**Age**, median (25th, 75th percentile)	45 (40, 48)	42 (37, 47)	59 (55, 63)	57 (53, 62)
**Recruitment site**				
Memorial Sloan Kettering Cancer Center	373 (67%)	431 (69%)	165 (59%)	223 (66%)
University of Pennsylvania Medical Center	65 (12%)	67 (11%)	44 (16%)	50 (15%)
University of Utah Huntsman Cancer Institute	115 (21%)	125 (20%)	73 (26%)	67 (20%)
**Menopausal status and reason**^a^				
Premenopausal	537 (97%)	597 (96%)		
Premenopausal (simple hysterectomy)	16 (3%)	26 (4%)		
Postmenopausal (natural)			212 (75%)	207 (61%)
Postmenopausal (oophorectomy)			25 (9%)	93 (27%)
Postmenopausal (simple hysterectomy)			38 (13%)	37 (11%)
Postmenopausal (reason unknown)			7 (3%)	3 (1%)
**Race/ethnicity**				
Non-Hispanic White	451 (81%)	513 (82%)	227 (80%)	301 (89%)
Non-Hispanic Black	31 (6%)	24 (4%)	17 (6%)	14 (4%)
Non-Hispanic Asian	31 (6%)	26 (4%)	15 (5%)	10 (3%)
Non-Hispanic (another race)	10 (2%)	16 (3%)	3 (1%)	3 (1%)
Hispanic (any race)	30 (5%)	44 (7%)	20 (8%)	12 (3%)
**Ever smoker**				
Never	426 (77%)	485 (78%)	194 (69%)	254 (75%)
Ever	127 (23%)	138 (22%)	88 (31%)	86 (25%)
**Body mass index at time of MRI**^b^ (kg/m^2^)				
< 25	346 (63%)	400 (64%)	113 (40%)	208 (61%)
25 to < 30	121 (22%)	143 (23%)	96 (34%)	67 (20%)
≥ 30	85 (15%)	78 (13%)	73 (26%)	64 (19%)
Unknown	1	2	0	1
**Parity**				
0	194 (35%)	230 (37%)	67 (24%)	83 (24%)
1	89 (15%)	77 (12%)	49 (17%)	50 (15%)
2+	270 (50%)	316 (51%)	166 (59%)	197 (61%)
**Family history of breast cancer**^c^				
No	399 (77%)	210 (34%)	181 (68%)	103 (31%)
Yes	116 (23%)	399 (66%)	85 (32%)	231 (69%)
Unknown (*n*)	38	14	16	6
***BRCA1*** **mutation**^d^				
Negative	340 (66%)	202 (33%)	99 (36%)	127 (39%)
Positive	9 (1.7%)	78 (13%)	4 (1.4%)	29 (8.8%)
Not tested	166 (32%)	329 (54%)	173 (63%)	173 (53%)
Unknown	38	14	6	11
***BRCA2*** **mutation**^d^				
Negative	340 (66%)	202 (33%)	95 (34%)	105 (32%)
Positive	9 (1.7%)	78 (13%)	8 (2.9%)	51 (16%)
Not tested	166 (32%)	329 (54%)	173 (63%)	173 (53%)
Unknown	38	14	6	11
**History of LCIS**				
No	546 (99%)	584 (94%)	274 (97%)	302 (89%)
Yes	7 (1%)	39 (6%)	8 (3%)	38 (11%)
**History of benign breast disease**				
No	443 (80%)	332 (53%)	210 (74%)	160 (47%)
Yes	110 (20%)	291 (47%)	72 (26%)	180 (53%)
MRIfibroglandular tissue				
Almost entirely fat				
Scattered FGT				
**Characteristics of cases**	***N*** **= 553**		***N*** **= 282**	
**Estrogen receptor**				
Negative	88 (16%)		59 (21%)	
Positive	462 (84%)		222 (79%)	
Unknown	3		1	
**Progesterone receptor**				
Negative	114 (21%)		91 (33%)	
Positive	436 (79%)		188 (67%)	
Unknown	3		3	
**HER2 expression**				
Negative	443 (82%)		236 (86%)	
Positive	95 (18%)		37 (14%)	
Unknown	15		9	
**Triple-negative**^e^ **subtype**				
No	483 (90%)		232 (85%)	
Yes	55 (10%)		41 (15%)	
Unknown	15		9	
**Histology**				
Ductal only	492 (90%)		226 (82%)	
Mixed ductal/lobular	6 (1%)		3 (1%)	
Lobular	43 (8%)		39 (14%)	
Other	5 (1%)		9 (3%)	
Unknown	7		5	
**Stage**				
Localized (breast only)	383 (69%)		214 (76%)	
Regional (breast and regional nodes)	162 (29%)		62 (22%)	
Distant	8 (2%)		4 (2%)	
Unknown	0		2	

*Abbreviation*: *LCIS* lobular carcinoma in situ, *HER2* human epidermal growth factor 2

^a^“Premenopausal” refers to women who report continued menstrual cycles; “premenopausal (simple hysterectomy”) refers to women whose menstrual cycles stopped after a simple hysterectomy and were under 50 years of age at time of MRI; “postmenopausal” refers to women who reported a natural stop of menstrual cycles; “postmenopausal (oophorectomy)” refers to women who underwent menopause due to a bilateral oophorectomy; “postmenopausal (simple hysterectomy) refers to women whose menstrual cycles stopped after a hysterectomy and were 50 years of age or older at the time of MRI; “postmenopausal (other)” refers to women whose menstrual cycle stopped due to medication or medical procedures or did not give further details

^b^Calculated using self-reported height (m) and weight (kg)

^c^Includes 1st-degree female relatives and any1st- or 2nd-degree male relative

^d^Self-reported by participants via structured questionnaire; variants of unknown significance were considered negative

^e^Triple-negative refers to women with tumors negative for estrogen receptors, progesterone receptors, and HER2 expression

The distribution of BPE differed by menopausal status (Table 2): 18% of premenopausal women had minimal BPE, while 44% of postmenopausal women had minimal BPE. There was no detectable correlation between BPE and FGT categories (*r* = 0.03, *p* = 0.2). Among the subset with repeat measurements (*n* = 130), we found good agreement for BPE (weighted *κ* = 0.73, 95% CI 0.65–0.82) and FGT (weighted *κ* = 0.83, 95% CI 0.–76–0.90).

**Table 2:** Distribution of fibroglandular tissue and background parenchymal enhancement in premenopausal and postmenopausal women in the IMAGINE study

	Premenopausal^**a**^ women	Postmenopausal^**a**^ women
**Fibroglandular tissue**^**b**^	**No. (%)**	**No. (%)**
Almost entirely fat	49 (4.2%)	78 (13%)
Scattered	225 (19%)	241 (39%)
Heterogeneous	598 (51%)	249 (40%)
Extreme	304 (26%)	54 (8.7%)
**Background parenchymal enhancement**^**b**^	**No. (%)**	**No. (%)**
Minimal	213 (18%)	275 (44%)
Mild	538 (46%)	265 (43%)
Moderate	306 (26%)	66 (11%)
Marked	119 (10%)	16 (2.6%)

^a^“Premenopausal” refers to women who report continued menstrual cycles and women whose menstrual cycles stopped after a simple hysterectomy and were under 50 years of age at time of MRI; “postmenopausal” refers to women who reported a natural stop of menstrual cycles, women who underwent menopause due to a bilateral oophorectomy, women whose menstrual cycles stopped after a hysterectomy and were 50 years of age or older at the time of MRI, and women whose menstrual cycle stopped due to medication or medical procedures or did not give further details

^b^Estimated for a single unaffected breast using BI-RADS (Breast Imaging - Reporting and Data System) guidelines by a single research radiologist blinded to case-control status, clinical characteristics, and medical history

In Table 3, we present the adjusted associations between BPE and breast cancer in multivariable logistic regression. Analysis using all four levels of BPE as the independent variable revealed a non-linear association between BPE and breast cancer risk, with large confidence intervals for the moderate/marked estimates in postmenopausal women. Therefore, we dichotomized BPE differentially for pre- and postmenopausal women based on previous studies and justified by the distribution of the data. Among premenopausal women, moderate/marked BPE was statistically significantly associated with breast cancer (OR = 1.49, 95% CI 1.05–2.11; *p* = 0.02). In postmenopausal women, mild/moderate/marked BPE had a suggestive but non-significant association with breast cancer (OR = 1.45, 95% CI 0.92–2.27; *p* = 0.10). A multiplicative interaction term between BPE and FGT did not improve the model fit for premenopausal women (likelihood ratio test *p* = 0.8) or postmenopausal women (*p* = 0.9). In Table 3, we also provide results from multivariable models stratified by BMI (< 25 vs ≥ 25). Among premenopausal women, the OR for moderate/marked BPE appeared to be higher among women with BMI ≥ 25 (OR 1.86, 95% CI 1.03–3.37; *p* = 0.04) compared to women with BMI < 25 (OR 1.32, 95% CI 0.84–2.03; *p* = 0.2). A similar pattern was observed for postmenopausal women (BMI ≥ 25: OR 2.20, 95% CI 1.02–4.71, *p* = 0.04; BMI < 25: OR 1.06, 95% CI 0.58–1.96, *p* = 0.8). Overall, BMI was associated with BPE, with 34% of women with BMI ≥ 25 having moderate/marked BPE compared to 25% of women with BMI < 25 (*p* < 0.001).

**Table 3:** Adjusted association between BPE and breast cancer in the IMAGINE study

**Premenopausal women**				
**BPE classification**^**a**^	**Cases**	**Controls**	**OR**^**b**^	**95% CI**
**4-level BPE**	*N* (%)	*N* (%)		
Minimal	84 (18%)	107 (18%)	Reference	
Mild	206 (43%)	287 (48%)	1.00	0.63–1.58
Moderate	138 (29%)	137 (23%)	1.72	1.03–2.88
Marked	49 (10%)	62 (10%)	1.00	0.51–1.94
**Dichotomous BPE**				
Minimal/mild BPE^c^	290 (61%)	394 (66%)	Reference	
Moderate/marked BPE	187 (39%)	199 (34%)	1.49	1.05–2.11
**Women with BMI < 25**^**d**^				
Minimal/mild BPE	197 (65%)	266 (70%)	Reference	
Moderate/marked BPE	106 (35%)	112 (30%)	1.31	0.84–2.03
**Women with BMI ≥ 25**				
Minimal/mild BPE	93 (53%)	128 (60%)	Reference	
Moderate/marked BPE	81 (47%)	87 (40%)	1.86	1.03–3.37
**Postmenopausal women**				
**BPE classification**	**Cases**	**Controls**	**OR**^**a**^	**95% CI**
Minimal	94 (36%)	162 (50%)	Reference	
Mild	125 (48%)	124 (39%)	1.42	0.89–2.28
Moderate	31 (12%)	30 (9%)	1.64	0.75–3.59
Marked	10 (4%)	6 (2%)	1.32	0.38–4.55
**Dichotomous BPE**				
Minimal BPE^c^	94 (36%)	162 (50%)	Reference	
Mild/moderate/marked BPE	166 (64%)	160 (50%)	1.45	0.92–2.27
**Women with BMI < 25**				
Minimal BPE	56 (52%)	116 (57%)	Reference	
Mild/moderate/marked BPE	52 (48%)	86 (43%)	1.06	0.58–1.96
**Women with BMI ≥ 25**				
Minimal BPE	38 (25%)	46 (38%)	Reference	
Mild/moderate/marked BPE	114 (75%)	74 (62%)	2.20	1.02–4.71

*Abbreviations*: *OR* odds ratio, *CI* confidence interval, *BMI* body mass index

^a^Estimated for a single unaffected breast using BI-RADS (Breast Imaging - Reporting and Data System) guidelines by a single research radiologist blinded to case-control status, clinical characteristics, and medical history

^b^ORs are estimated in a multivariable conditional logistic regression model with adjustment for FGT (heterogeneous/dense vs fatty/scattered); history of simple hysterectomy; BMI (< 25, ≥ 25 and < 30, ≥ 30 kg/m¬2); parity; 1st-degree female family history or 1st- or 2nd-degree male family history of breast cancer; BRCA testing history; presence of BRCA mutations; history of lobular carcinoma in situ (LCIS); history of benign breast disease; and conditioned on matching criteria: race/ethnicity (non-Hispanic White vs other), recruitment site, and age at MRI (5-year categories)

^c^Parameterization of BPE differs for premenopausal and postmenopausal women to capture differing distributions of BPE in these groups

^d^Models stratified by BMI at the time of MRI

In sensitivity analyses, we repeated the primary analysis restricting to non-Hispanic White women and found that the results were similar. Additionally, we restricted to matched case-control image pairs read in the same batch and estimated the association between breast cancer and BPE conditional on matched pair strata; these results were likewise not materially changed compared to the primary analysis. Finally, we excluded women who reported a history of simple hysterectomy, for whom it was not possible to determine the timing of menopause, and the results were also unchanged (Supplementary Table 2).

Restricting to cases, HER2 overexpression of the tumor was associated with BPE in the unaffected breast (OR = 1.72, 95% CI 1.07–2.76; *p* = 0.03) among premenopausal women, and hormone receptor (ER and/or PR) positivity was associated with BPE in the unaffected breast among postmenopausal women (OR 2.55, 95% CI 1.31–5.34; *p* = 0.01) (Table 4).

**Table 4:** The association between BPE and tumor characteristics among women with breast cancer in the IMAGINE study

**Premenopausal cases**				
**Tumor characteristic**	**Minimal/mild BPE**	**Moderate/marked BPE**^**a**^	**OR**^**b**^	**95% CI**
**Hormone receptor status**^**c**^				
Negative	48 (15%)	33 (16%)	Reference	
Positive	277 (85%)	178 (84%)	1.00	0.59–1.68
**HER2**				
Negative	277 (85%)	164 (78%)	Reference	
Positive	48 (15%)	47 (22%)	1.72	1.07–2.76
**Histology**				
Ductal	293 (90%)	194 (92%)	Reference	
Lobular/mixed/other	32 (10%)	17 (8%)	0.99	0.52–1.91
**Stage**				
Localized	220 (68%)	147 (70%)	Reference	
Regional	101 (31%)	60 (28%)	0.86	0.57–1.29
Distant	4 (1%)	4 (2%)	1.45	0.34–6.21
**Postmenopausal cases**				
**Tumor characteristic**	**Minimal BPE**	**Mild/moderate/marked BPE**	**OR**^**b**^	**95% CI**
**Hormone receptor status**				
Negative	26 (27%)	30 (17%)	Reference	
Positive^c^	71 (73%)	145 (83%)	2.55	1.22–5.34
**Human epidermal growth factor 2**				
Negative	83 (86%)	152 (87%)	Reference	
Positive	14 (14%)	23 (13%)	0.89	0.37–2.13
**Histology**				
Ductal	80 (82%)	151 (86%)	Reference	
Lobular/mixed/other	17 (18%)	24 (14%)	0.65	0.30–1.43
**Stage**				
Localized	76 (78%)	132 (75%)	Reference	
Regional	19 (20%)	41 (23%)	0.75	0.36–1.53
Distant	2 (2%)	2 (1%)	0.36	0.04–3.17

*Abbreviations*: *OR* odds ratio, *CI* confidence interval, *ER* estrogen receptor, *PR* progesterone receptor, *HER2* human epidermal growth factor 2

^a^Estimated for a single unaffected breast using BI-RADS (Breast Imaging - Reporting and Data System) guidelines by a single research radiologist blinded to case-control status, clinical characteristics, and medical history

^b^Multivariable-adjusted odds ratio for BPE category estimated in logistic regression with additional adjustment for (heterogeneous/dense vs fatty/scattered); BMI (< 25, ≥25 and < 30, ≥ 30 kg/m^2^); and conditioned on matching criteria: race/ethnicity (non-Hispanic White vs other), recruitment site, and age at MRI (5-year categories)

^c^Considered positive if positive for estrogen or progesterone receptors

## Discussion

Among premenopausal women, moderate or marked BPE was associated with a 49% increased odds of breast cancer relative to controls, which was robust in our sensitivity analyses. Among postmenopausal women, our results suggest that any BPE above minimal may be associated with increased risk of breast cancer. Additionally, our exploratory analysis stratified by BMI suggests possible effect modification of the association between BPE and breast cancer by BMI for both premenopausal and postmenopausal women. This finding is plausible given the possible relationship between visceral adipose tissue mass, endogenous hormone production, and BPE [28], but further study in distinct populations are needed to clarify the relationship between BMI, BPE, and breast cancer.

To our knowledge, there are eight previous studies that have tested the association between qualitative BPE and breast cancer (summarized in Table 5). Estimates of the BPE-breast cancer association were based on several different study designs of varying sizes (*N* = 26 to *N* = 4247) and ranged from a non-significant OR of 1.2 (95% CI 0.5–3.3) [18] for comparing malignant to benign lesions, to a significant OR of 7.7 (95% CI 1.5–39.5) for comparing breast cancer cases to cancer-free controls. Although most studies found a positive association between BPE and breast cancer, all had one or more limitations that precluded clear interpretation: (A) incomplete blinding of clinical characteristics including case/control status; (B) assessment of BPE with view of the affected breast; and/or (C) no accounting for menopausal status, which strongly affects BPE and the risk of breast cancer. Most importantly, assessment of BPE without blinding to the affected breast would likely bias the measures of association between BPE and breast cancer away from the null. For example, without blinding of the affected breast, Telegrafo et al. reported that 0/224 (0%) of controls had marked BPE, compared to 72/162 (44%) of invasive cases, corresponding to an unadjusted odds ratios as high as 61 for moderate/marked vs minimal/mild BPE [19]. This distribution of BPE is not consistent with the larger and more representative data from the IMAGINE study or that of Arasu et al., which used pre-diagnosis MRI assessments from the Breast Cancer Surveillance Consortium [29]. The longitudinal study by Arasu et al. is well designed, using clinical BPE assessments among cancer-free women and following until a diagnosis of breast cancer, which ensured that the BPE assessment was not biased by presence of breast cancer. Inter-observer variability of BPE assessment in the study by Arasu et al. adds uncertainty into the effect of the association between BPE and breast cancer, but also more closely represents current clinical practice. On the other hand, our single reader approach proved to be reliable and eliminated inter-observer variability, providing strong internal validity to estimate the strength of association between BPE and breast cancer. Our measures of association, although elevated, were more modest than previous work. It is plausible that complete blinding of MRI assessment reduced bias in BPE assessment and thereby attenuated the measure of association relative to previous unblinded studies.

**Table 5:** Summary of existing studies reporting associations between background parenchymal enhancement (BPE) and breast cancer

Author	Number of breast cancer cases	Number of comparison women	MRI assessment methods		Association between BPE and breast cancer		
			Blind to clinical data	Blind to affected breast	Pre-menopausal	Post-menopausal	Overall
**Arasu**^a^ **et al.** [16, 29]	129 (invasive) 47 (DCIS)	4071 (cancer-free)	No	N/A^a^	HR 3.0^b^ (1.3–7.1)	HR 2.6^b^ (1.4–4.6)	HR 2.3^b^ (1.5–3.4)
**Grimm et al.** [17]	43 (invasive) 18 (DCIS)	122 (cancer-free)	Yes	No			OR 2.5^b^ (1.3–4.8)
**Melsaether et al.** [20]	81 (invasive) 35 (DCIS)	116 (cancer-free)	Yes	No			2 readers at 3 time points (range: OR 1.0 to 7.7)^,e^
**Bennani-Baiti et al.** [18]	353 (invasive)	187 (benign)	Yes	No			OR 1.2^d^ (0.5–3.3)
**Telegrafo et al.** [19]	78 (invasive)	52 (benign) 50 (negative)	Yes	No			
**Dontchos et al.**	12 (invasive) 11 (DCIS)	23 (cancer-free)	Yes	No			OR 9.0^b^ (1.1–71.0)
**Albert et al.** [21]	294 (invasive) 104 (in situ)	72 (cancer-free)	Yes	No	^f^	^f^	
**King et al.** [14]	25 (invasive) 14 (DCIS)	78 (cancer-free)	No	No	OR 2.2^c^ (0.4–11.6)	OR 4.1^c^ (1.3–13.2)	OR 3.3^c^ (1.3–8.3)

*Abbreviations*: *DCIS* ductal carcinoma in situ, *HR* hazards ratio, *OR* odds ratio

^a^Cohort study. All others are case-control studies

^b^Comparing mild/moderate/marked vs minimal BPE

^c^Comparing moderate/marked vs minimal/mild BPE

^d^OR for a 1-unit increase in BPE

^e^Comparing controls that later developed breast cancer (*n* = 9) to those that did not develop breast cancer (107)

^f^*P* value for age-adjusted association between BPE and breast cancer was 0.15 for premenopausal women and < 0.001 for postmenopausal women. However, direction of association is not given and data provided do not permit calculation of ORs

There is a biologically plausible relationship between increased BPE and breast cancer. A previous histopathological study demonstrated that elevated BPE was associated with greater microvascular concentration and expression of vascular endothelial growth factor [30], suggesting that BPE is a marker of increased concentrations of glandular tissue within FGT. However, significant histopathological associations were limited to premenopausal women. Therefore, the histopathologic evidence of a biological relationship between BPE and glandular tissue concentration combined with the growing epidemiological evidence of an association between BPE and breast cancer suggests that BPE is a new biomarker of breast cancer, at least for premenopausal women.

We also reported associations between BPE and tumor characteristics among cases. There are limited previous studies that have evaluated the relation between breast tumor histology and BPE. In our study, HER2-positive cancer was independently associated with higher BPE in the unaffected breast among premenopausal cases. To our knowledge, no studies have identified a positive association between HER2-overexpression and BPE in the unaffected breast, although at least one has reported an association between triple-negative breast cancer and increased BPE [31]. We also found that the hormone receptor status of tumors was independently associated with BPE in the unaffected breast among postmenopausal cases. Two small clinical studies previously identified univariable associations between hormone receptors and BPE [32, 33] while others did not identify any associations [34–36]. This study is the first to report the association in a large population of women with breast cancer using a multivariable approach, but these results should be interpreted with caution and require confirmation in future studies.

This study had a number of strengths. The IMAGINE study was designed specifically to assess the association between BPE and breast cancer for premenopausal and postmenopausal women with centralized reading of MRIs. The centralized reading of MRIs eliminated inter-observer variability and improved our internal validity. We also found that our single-reader design provided good intra-observer variability, but the single-reader approach may nonetheless reduce external generalizability. High intra- and inter-observer variability of visual assessment of BPE have been reported in previous studies [37], justifying the development of fully automated, objective measures of BPE for eventual clinical implementation of BPE in risk prediction. In addition, our study radiologist was blinded to both case-control status and clinical characteristics of patients and assessed BPE only in the unaffected breast without view of the affected breast, which resulted in good intra-observer reliability consistent with previous studies [38]. Furthermore, the study included a large population of cases and unaffected controls, allowing for subgroup analysis by menopausal status and a case-only evaluation of tumor subtypes.

Nonetheless, there are limitations which should be considered. First, as the source population was women undergoing breast MRI, the control group included many “high-risk” women undergoing routine screening for breast cancer, whereas the cases primarily received MRI as part of a diagnostic workup. As a result, the control population had a greater number of women with breast cancer risk factors. With adjustment for these factors, we accounted for the differing distribution of these factors as well as their association with breast cancer, but residual confounding due to selection bias is possible. Any residual confounding would bias the association between breast cancer and BPE to the null, reducing the probability of a false-positive finding. Second, this study did not have detailed information about MRI acquisition, which differed somewhat among participating recruitment sites. However, all participating sites are National Cancer Institute-designated Comprehensive Cancer Centers following American College of Radiology guidelines, and the analysis of MRI series with differing acquisition settings improves clinical generalizability. Third, this study lacked power to assess the utility of BPE in specific minority racial/ethnic groups, but our sensitivity analysis showed that the results were similar overall and when limited to non-Hispanic White women.

## Conclusions

We found that BPE is consistently associated with breast cancer among premenopausal women and may be associated with breast cancer among postmenopausal women. Our findings confirm results from previous high-quality studies describing this association and the results are generalizable to women undergoing breast MRI, who, at present, are primarily women at high risk of breast cancer. BPE assessed by a radiologist is still limited by intra- and inter-observer variability, which may explain the unexpected non-linear association between BPE and breast cancer found among premenopausal women. As the use of MRI increases and is adopted in more settings [39, 40], future studies are needed to (a) develop objective and reproducible measures of BPE and (b) evaluate the ability of BPE to improve risk prediction for breast cancer after accounting for known risk factors. Fully automated methods of BPE assessment would permit the implementation of reproducible risk prediction in routine clinical settings, allowing the personalization breast cancer screening recommendations to improve early detection and reduce harms associated with overscreening.

## Supplementary Information

**Additional file 1: Table S1.** Characteristics of participants that were successfully matched to those that were not successfully matched. This table provides a comparison of the characteristics of women that were successfully matched (*N* = 1630) and those that were not successfully matched (*N* = 168). **Table S2.** Additional analyses of the association between background parenchymal enhancement and breast cancer in the Imaging and Epidemiology (IMAGINE) Study. This table displays the results of our additional multivariable analysis: (1) restricting to non-Hispanic White women, (2) using matched conditional logistic regression restricting to women that were successfully matched, and (3) excluding women with a history of simple hysterectomy, for whom timing of menopause is unclear. The results in each of the additional analyses were not markedly different from the primary analysis.

## Data Availability

The datasets generated during and/or analyzed during the current study are available from the corresponding author on reasonable request.

## Abbreviations

FGT: Fibroglandular tissue
MRI: Magnetic resonance imaging
BPE: Background parenchymal enhancement
BI-RADS: Breast Imaging Reporting and Data System
IMAGINE: Imaging and Epidemiology Study
DCIS: Ductal carcinoma in situ
LCIS: Lobular carcinoma in situ
BMI: Body mass index
HER2: Human epidermal growth factor 2
IQR: Interquartile range
