# Supplementary material for: Association of breast cancer with MRI background parenchymal enhancement: the IMAGINE case-control study
Source: Breast Cancer Res. 2020 Dec 7;22:138. doi: 10.1186/s13058-020-01375-7 (PMC7722419; doi:10.1186/s13058-020-01375-7)
Supplement: Supplementary file 1 — Additional file 1: Table S1. Characteristics of participants that were successfully matched to those that were not successfully matched. This table provides a comparison of the characteristics of women that were successfully matched (N = 1630) and those that were not successfully matched (N = 168). Table S2. Additional analyses of the association between background parenchymal enhancement and breast cancer in the Imaging and Epidemiology (IMAGINE) Study. This table displays the results of our additional multivariable analysis: (1) restricting to non-Hispanic White women, (2) using matched conditional logistic regression restricting to women that were successfully matched, and (3) excluding women with a history of simple hysterectomy, for whom timing of menopause is unclear. The results in each of the additional analyses were not markedly different from the primary analysis. [file 13058_2020_1375_MOESM1_ESM.docx]

**Supplementary Table 1.** Characteristics of participants that were successfully matched to those that were not successfully matched

| **Characteristic** | **Matched**,  N = 1630 | **Not Matched,**  N = 168 |
| --- | --- | --- |
| **Case-control status** |  |  |
| Control | 815 (50%) | 148 (88%) |
| Case | 815 (50%) | 20 (12%) |
| **Age at time of MRI** |  |  |
| <45 years | 596 (37%) | 89 (53%) |
| 45 to <60 years | 790 (48%) | 68 (40%) |
| ≥60 years | 244 (15%) | 11 (6.5%) |
| **Recruitment site** |  |  |
| Memorial Sloan Kettering Cancer Center | 1056 (65%) | 136 (81%) |
| University of Pennsylvania Medical Center | 202 (12%) | 24 (14%) |
| University of Utah Huntsman Cancer Institute | 372 (23%) | 8 (4.8%) |
| **Menopausal status and reason** |  |  |
| Premenopausal*^a^* | 1035 (63%) | 99 (59%) |
| Premenopausal (Simple hysterectomy) | 36 (2.2%) | 6 (3.6%) |
| Postmenopausal (Natural) | 378 (23%) | 41 (24%) |
| Postmenopausal (Oophorectomy) | 99 (6.1%) | 19 (11%) |
| Postmenopausal (Simple hysterectomy) | 72 (4.4%) | 3 (1.8%) |
| Postmenopausal (Reason Unknown) | 10 (0.6%) | 0 (0%) |
| **Race/Ethnicity** |  |  |
| Non-Hispanic White | 1341 (82%) | 151 (90%) |
| Not non-Hispanic White | 289 (18%) | 17 (10%) |
| **Ever smoker** |  |  |
| Never | 1235 (76%) | 124 (74%) |
| Ever | 395 (24%) | 44 (26%) |
| **Body at mass index at time of MRI***^b^* |  |  |
| <25 | 955 (59%) | 112 (67%) |
| 25 to <30 | 388 (24%) | 39 (23%) |
| ≥30 | 283 (17%) | 17 (10%) |
| **Parity** |  |  |
| Nulliparous | 499 (31%) | 75 (45%) |
| 1 | 239 (15%) | 26 (15%) |
| 2+ | 892 (55%) | 67 (40%) |
| **Family history of breast cancer***^c^* |  |  |
| No | 832 (53%) | 61 (37%) |
| Yes | 729 (47%) | 102 (63%) |
| Unknown | 69 | 5 |
| ***BRCA1* mutation***^d^* |  |  |
| Negative | 717 (46%) | 60 (37%) |
| Positive | 81 (5.2%) | 30 (18%) |
| Not Tested | 768 (49%) | 73 (45%) |
| Unknown | 64 | 5 |
| ***BRCA2* mutation***^d^* |  |  |
| Negative | 678 (43%) | 64 (39%) |
| Positive | 120 (7.7%) | 26 (16%) |
| Not Tested | 768 (49%) | 73 (45%) |
| Unknown | 64 | 5 |
| **History of LCIS** |  |  |
| No | 1546 (95%) | 160 (95%) |
| Yes | 84 (5.2%) | 8 (4.8%) |
| **History of benign breast disease** |  |  |
| No | 1045 (64%) | 100 (60%) |
| Yes | 585 (36%) | 68 (40%) |
| **Fibroglandular tissue on MRI** |  |  |
| Fatty | 117 (7.2%) | 10 (6.0%) |
| Scattered | 427 (26%) | 39 (23%) |
| Heterogeneous | 771 (47%) | 76 (45%) |
| Dense | 315 (19%) | 43 (26%) |
| **Background parenchymal enhancement on MRI** |  |  |
| Minimal | 422 (26%) | 66 (39%) |
| Mild | 731 (45%) | 72 (43%) |
| Moderate | 348 (21%) | 24 (14%) |
| Marked | 129 (7.9%) | 6 (3.6%) |

**Abbreviation.** LCIS, lobular carcinoma *in situ*; HER2, human epidermal growth factor 2.

*^a^* “Premenopausal” refers to women who report continued menstrual cycles; “Premenopausal (Simple Hysterectomy”) refers to women whose menstrual cycles stopped after a simple hysterectomy and were under 50 years of age at time of MRI; “Postmenopausal” refers to women who reported a natural stop of menstrual cycles; “Postmenopausal (Oophorectomy)” refers to women who underwent menopause due to a bilateral oophorectomy; “Postmenopausal (Simple hysterectomy) refers to women whose menstrual cycles stopped after a hysterectomy and were 50 years of age or older at the time of MRI; “Postmenopausal (Other)” refers to women whose menstrual cycle stopped due to medication or medical procedures or did not give further details.

*^b^* Calculated using self-reported height (m) and weight (kg)

*^c^* Includes first-degree female relatives and any1st- or 2^nd^-degree male relative.

*^d^* Self-reported by participants via structured questionnaire; variants of unknown significance were considered negative.


**Supplementary Table 2. Additional analyses of the association between background parenchymal enhancement and breast cancer in the Imaging and Epidemiology (IMAGINE) Study**

|  | **Cases** | **Controls** | **OR^a^** | **95% CI** |
| --- | --- | --- | --- | --- |
| **Restricting to non-Hispanic White women** | | | |  |
| **Premenopausal women** | | | |  |
| Background Parenchymal Enhancement^b^ |  |  |  |  |
| Minimal/Mild | 236 | 327 | Reference |  |
| Moderate/Marked | 158 | 165 | 1.55 | 1.06 - 2.27 |
| **Postmenopausal women** | | | |  |
| Background Parenchymal Enhancement |  |  |  |  |
| Minimal | 82 | 144 | Reference |  |
| Mild, Moderate or Marked | 132 | 139 | 1.29 | 0.80 - 2.10 |
|  | **Cases** | **Controls** | **OR^a^** | **95% CI** |
| **Matched conditional logistic regression** | | | |  |
| **Premenopausal women** | | | |  |
| Background Parenchymal Enhancement^b^ |  |  |  |  |
| Minimal/Mild | 280 | 330 | Reference |  |
| Moderate/Marked | 184 | 178 | 1.68 | 1.01 - 2.79 |
| **Postmenopausal women** | | | |  |
| Background Parenchymal Enhancement |  |  |  |  |
| Minimal | 91 | 133 | Reference |  |
| Mild, Moderate or Marked | 164 | 134 | 1.25 | 0.66 - 2.37 |
|  | **Cases** | **Controls** | **OR^a^** | **95% CI** |
| **Exclude women with history of simple hysterectomy** | | | |  |
| **Premenopausal women** | | | |  |
| Background Parenchymal Enhancement^b^ |  |  |  |  |
| Minimal/Mild | 282 | 383 | Reference |  |
| Moderate/Marked | 184 | 185 | 1.60 | 1.13 - 2.28 |
| **Postmenopausal women** |  |  |  |  |
| Background Parenchymal Enhancement |  |  |  |  |
| Minimal/Mild | 83 | 149 | Reference |  |
| Mild, Moderate or Marked | 144 | 142 | 1.54 | 0.96 - 2.47 |

**Abbreviations**. OR, odds ratio; CI, confidence interval

^a^ ORs are estimated in a multivariable conditional logistic regression model with adjustment for FGT (heterogenous/dense vs fatty/scattered); history of simple hysterectomy; BMI (<25, ≥25 and <30, ≥30 kg/m^2^); parity; first-degree female family history or 1st- or 2nd-degree male family history of breast cancer; BRCA testing history; presence of BRCA mutations; history of lobular carcinoma in situ (LCIS); history of benign breast disease; and conditioned on matching criteria: race/ethnicity (non-Hispanic White vs other), recruitment site, and age at MRI (5-year categories)

^b^ Parameterization of BPE differs for pre- and postmenopausal women to capture differing distributions of BPE in these groups

^c^ ORs are estimated in a multivariable conditional logistic regression model with adjustment for FGT (heterogenous/dense vs fatty/scattered); history of simple hysterectomy; BMI (<25, ≥25 and <30, ≥30 kg/m¬2); parity; first-degree female family history or 1st- or 2nd-degree male family history of breast cancer; BRCA testing history; presence of BRCA mutations; history of lobular carcinoma in situ (LCIS); history of benign breast disease; and conditioned on case-control strata.
